# Does adding thoracic radiation therapy to systemic chemotherapy increase 1-year and 2-year overall survival in patients with extensive-stage small-cell lung cancer? meta-analysis

**DOI:** 10.1186/s43046-025-00271-5

**Published:** 2025-04-29

**Authors:** Yasir A. Taha

**Affiliations:** Internal Medicine department, College of Medicine, Najran University, Najnan, KSA Saudi Arabia

**Keywords:** Extensive-stage small-cell lung cancer, Thoracic radiation therapy, Chemotherapy

## Abstract

**Background:**

Lung cancer is the leading cause of cancer-related mortality worldwide. Approximately 15–20% of newly diagnosed individuals with primary lung cancer have small cell lung cancer, and more than 60% of patients have advanced-stage small cell lung cancer at the time of diagnosis. Patients with advanced-stage small-cell lung cancer may benefit from thoracic radiation therapy.

This comprehensive meta-analysis was conducted to determine whether adding thoracic radiation to systemic chemotherapy increases 1-year and 2-year survival in patients with advanced-stage small-cell lung cancer.

**Methods:**

The Science Direct, PubMed, Embase, and Wanfang databases were comprehensively searched from 1980 to 2022. The inclusion criteria for studies were as follows: (1) all patients had advanced-stage small cell lung cancer; (2) a group receiving thoracic radiation therapy combined with chemotherapy was compared with a group receiving only chemotherapy; and (3) 1-year and 2-year overall survival data were provided. Pooled relative risks (RRs) and risk differences (RDs) were calculated, publication bias was evaluated, and sensitivity analysis was conducted.

**Results:**

Ten studies met the inclusion criteria. These studies included 922 patients (534 patients in the chemotherapy combined with thoracic radiation therapy (ChT/TRT) group and 388 patients in the chemotherapy (ChT) group).

The results of the meta-analysis revealed that the addition of thoracic radiotherapy to chemotherapy increased the 1-year overall survival rate to 52%, whereas the 1-year overall survival rate was 32.2% when chemotherapy alone was used. The addition of thoracic radiotherapy to chemotherapy also increased the 2-year survival rate to 18.7%, compared with 10% in the ChT group.

The ChT/TRT group had a significantly better 1-year overall survival rate than the ChT group, with a pooled RR of 1.61 (95% CI, 1.36–1.90, *P* < 0.00001) and a pooled RD of 0.2 (95% CI, 0.13–0.26, *P* < 0.00001).

The ChT/TRT group also had a significantly better 2-year overall survival rate than the ChT group, with a pooled RR of 1.90 (95% CI, 1.34–2.68, *P* = 0.0003) and a pooled RD of 0.09 (95% CI, 0.05–0.13, *P* < 0.0001).

**Conclusion:**

This study revealed that adding thoracic radiation therapy to chemotherapy increases both 1-year and 2-year survival in patients with extensive-stage small-cell lung cancer.

## Introduction

Small-cell lung cancer accounts for 10–15% of lung cancer cases. Small-cell lung cancer usually has an aggressive clinical course, with early metastatic dissemination, rapid tumor development, and frequent involvement of the liver and brain [1]. It is believed that extensive-stage small-cell lung cancer is a resistant carcinoma that progresses exceptionally quickly. Radiation therapy (RT), including thoracic radiation therapy (TRT) and brain radiation therapy (BRT), is beneficial for improving survival and for local management in extensive-stage small-cell lung cancer [2–5]. Controlling intrathoracic tumors is still a significant challenge for this disease. Previous studies have revealed a notable increase in local control and survival among extensive-stage small-cell lung cancer patients who received thoracic radiation therapy [6–8].

## The aim of this meta-analysis

The aim of this meta-analysis was to pool data from randomized clinical studies to determine the differences between patients with extensive-stage small cell lung cancer treated with systemic chemotherapy alone and those treated with systemic chemotherapy combined with thoracic radiation therapy.

## Material and methods

### Search strategy

The PubMed, Embase, Science Direct, and Wanfang databases were searched from 1980 to 2022 to identify studies examining the addition of thoracic radiotherapy to chemotherapy for the treatment of patients with small-cell lung cancer. The following keywords were used to search the databases: lung cancer, small-cell lung cancer, extensive-stage small-cell lung cancer, thoracic radiation therapy, and chemoradiotherapy. In order to mitigate the risk of publication bias, the reference lists of the retrieved studies were searched to identify additional eligible publications.

### Inclusion criteria

There were no restrictions regarding the patients’ age, sex, weight loss, location of distant metastases, performance status, or receipt of PCI. The inclusion criteria for studies were as follows:

1. All patients had extensive-stage small-cell lung cancer;
2. Two groups—one receiving chemotherapy combined with thoracic radiation therapy (ChT/TRT) and the other receiving only ChT (control group)—were compared.
3. The study included sufficient data to calculate 1- and 2-year overall survival rates.

Studies without control groups, case reports, and abstracts were not eligible for inclusion.

### Data extraction and outcomes

**Fig. 1:**
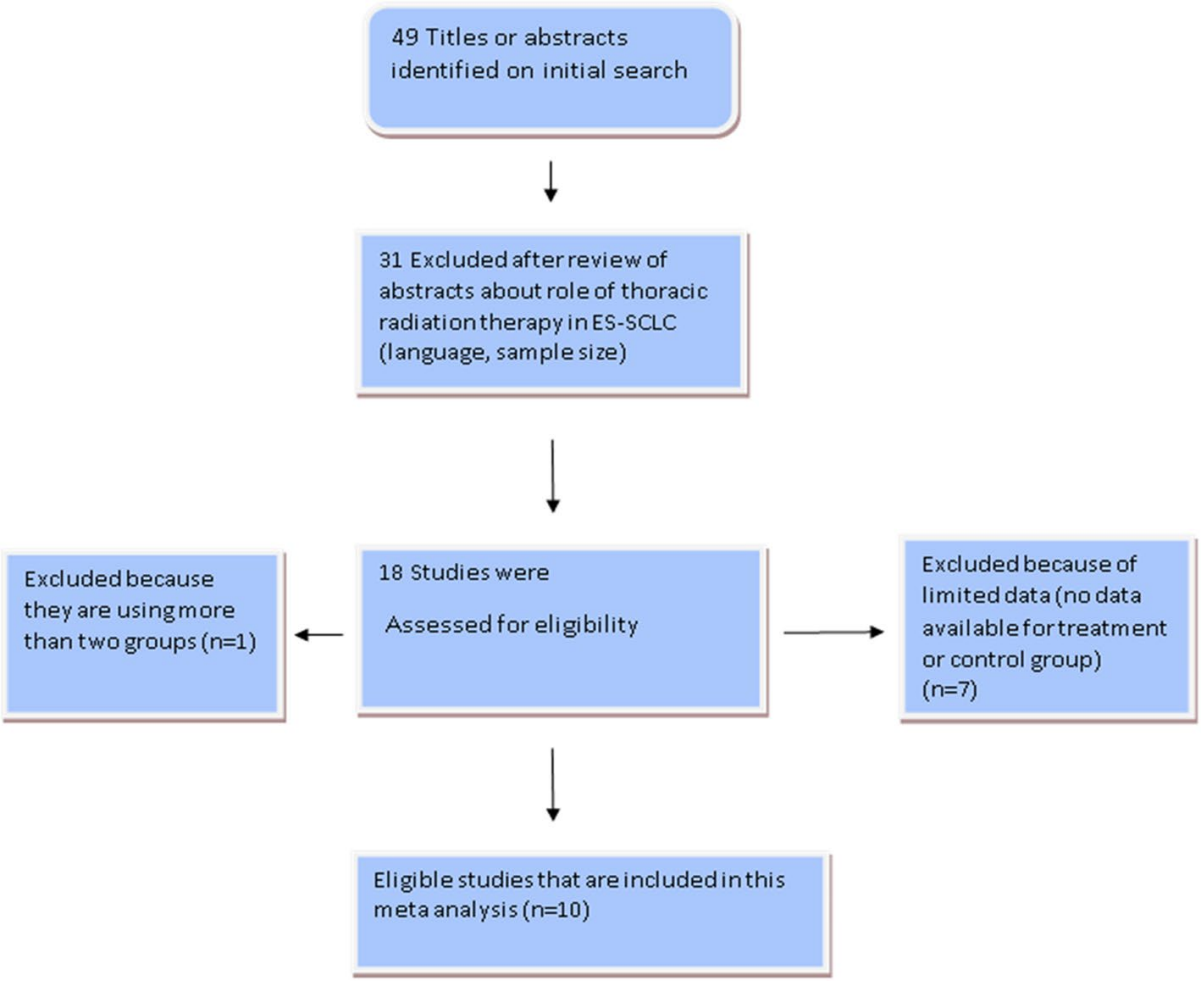
Flow diagram of study selection process for this meta-analysis

**Fig. 2:**
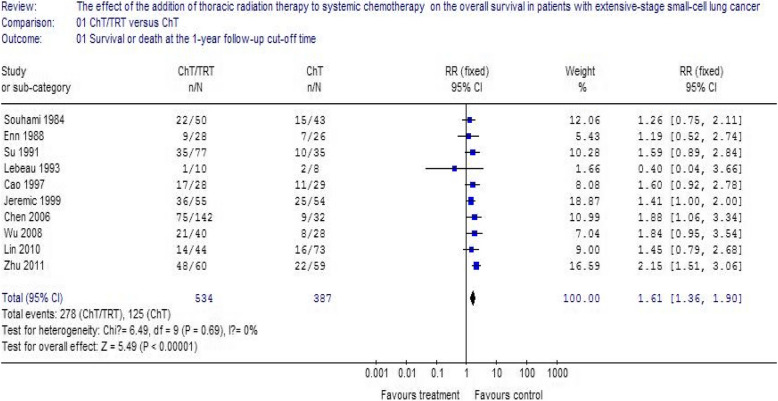
Forest plot of the survival at the 1-year follow-up cut-off time regarding the relative risk

**Fig. 3:**
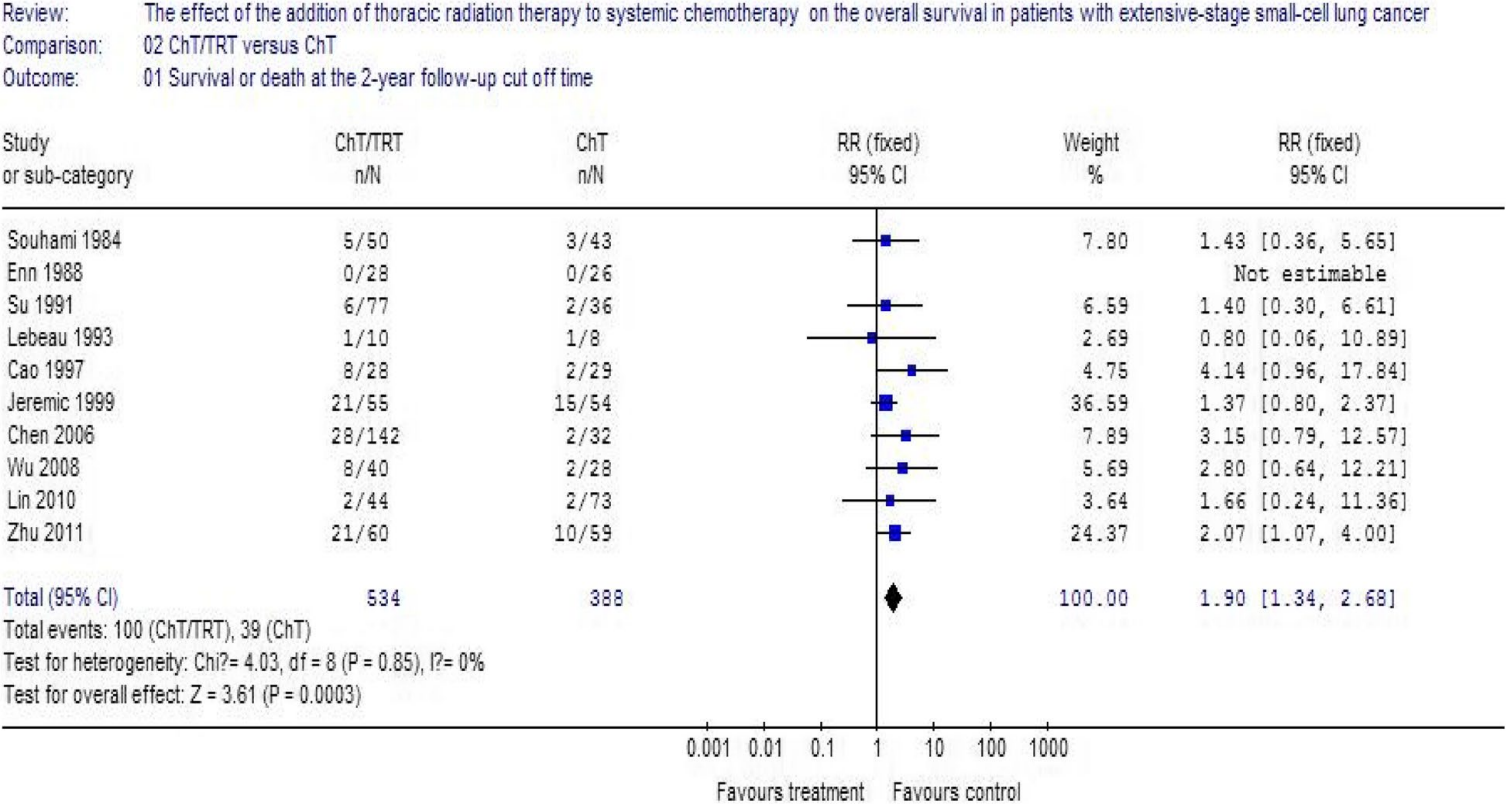
Forest plot of the survival at the 2-years follow-up cut-off time regarding the relative risk

**Fig. 4:**
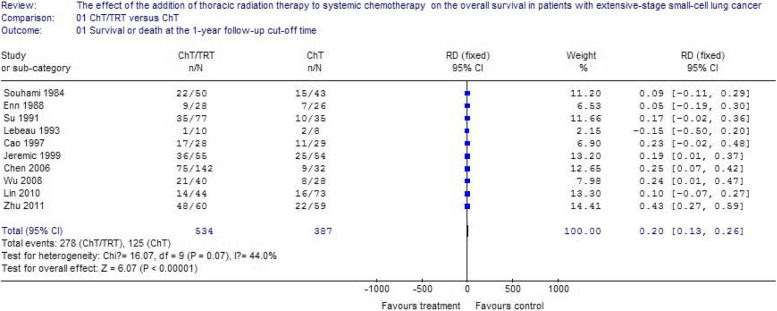
Forest plot of the survival at the 1-year follow-up cut-off time regarding the risk difference

**Fig. 5:**
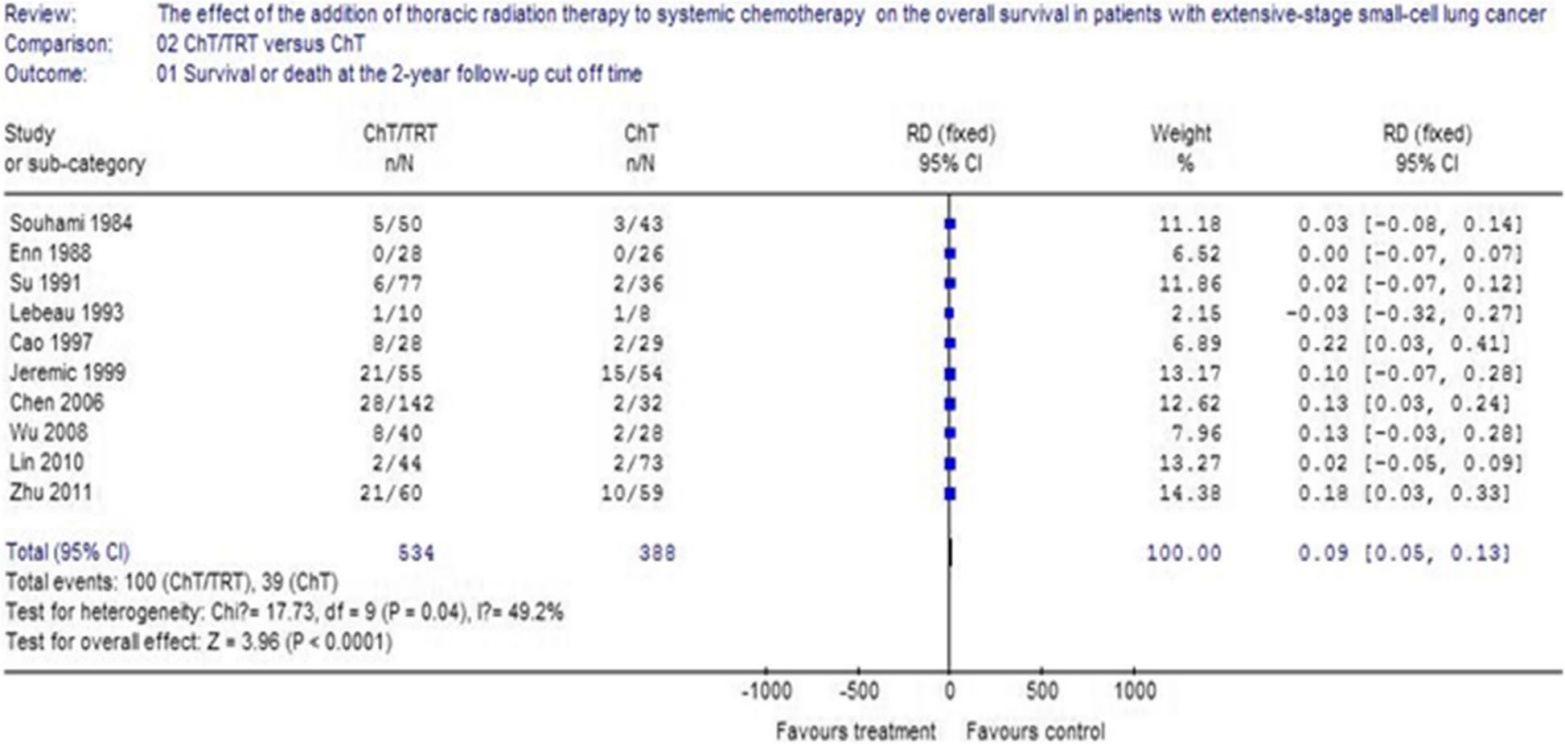
Forest plot of the survival at the2-year follow-up cut-off time regarding the risk difference

**Fig. 6:**
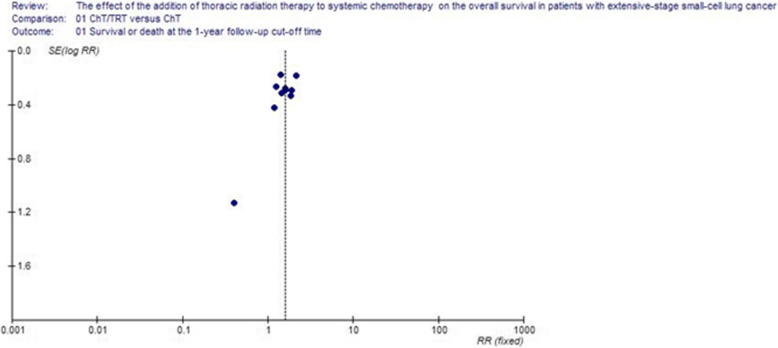
Funnel plot of the survival at the 1-year follow-up cut-off time regarding the relative risk

**Fig. 7:**
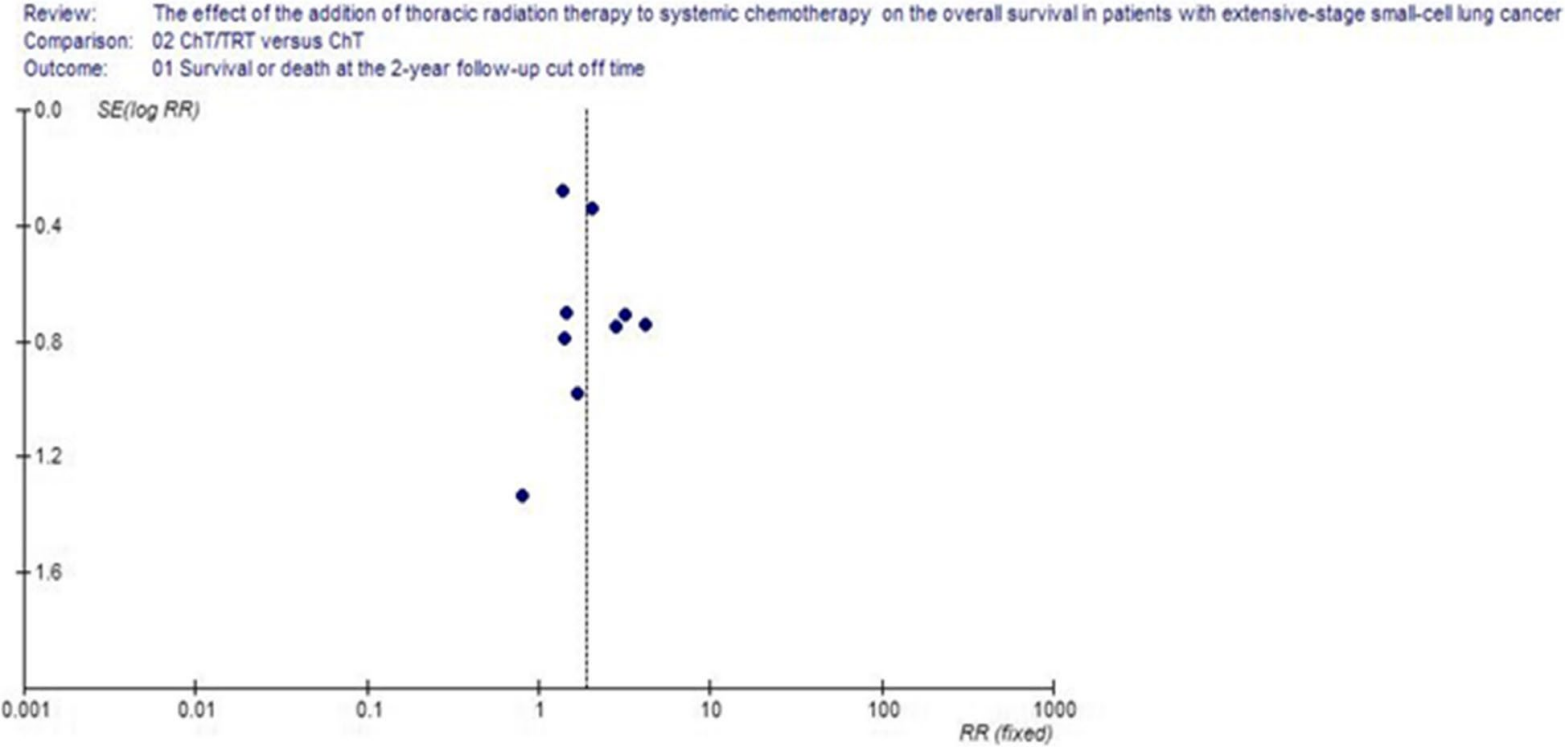
Funnel plot of the survival at the 2-year follow-up cut-off time regarding the relative risk

**Fig. 8:**
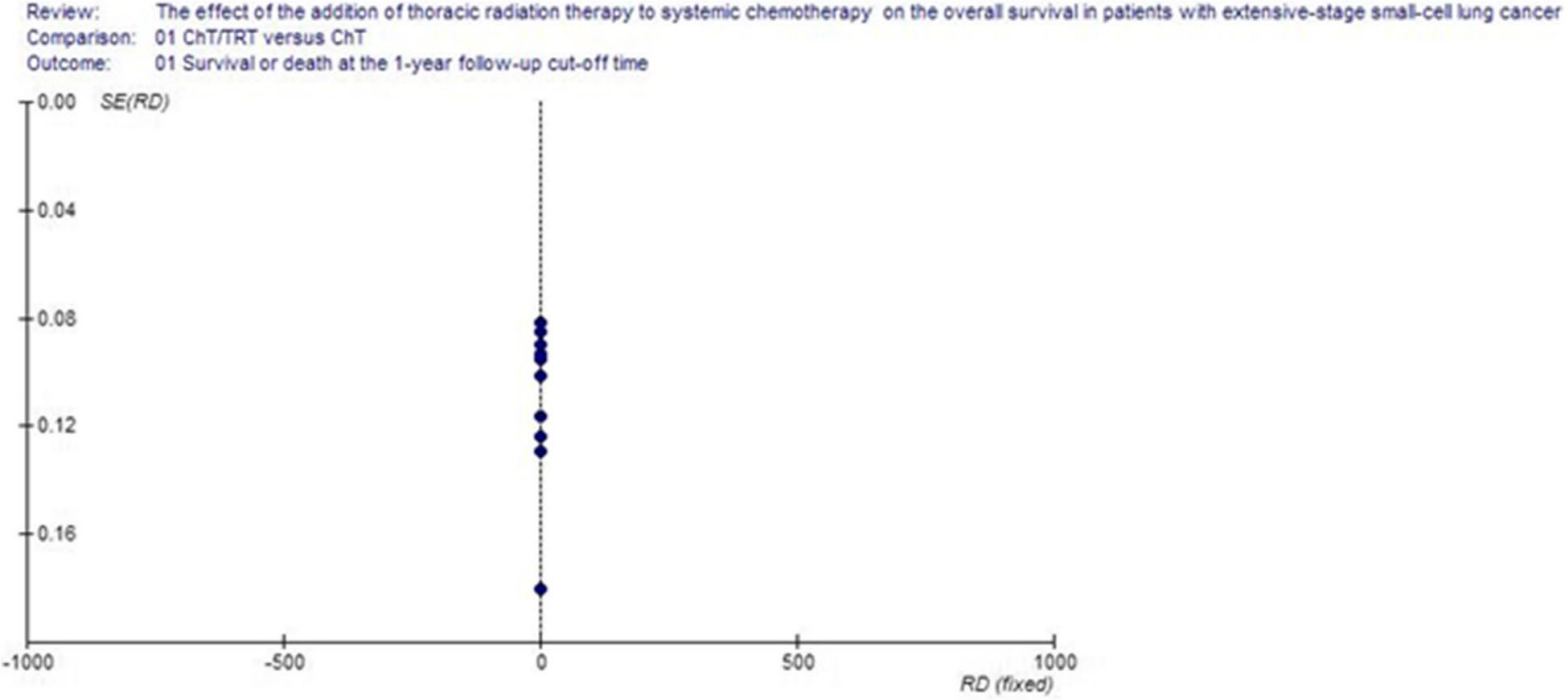
Funnel plot of the survival at the 1-year follow-up cut-off time regarding the risk difference

**Fig. 9:**
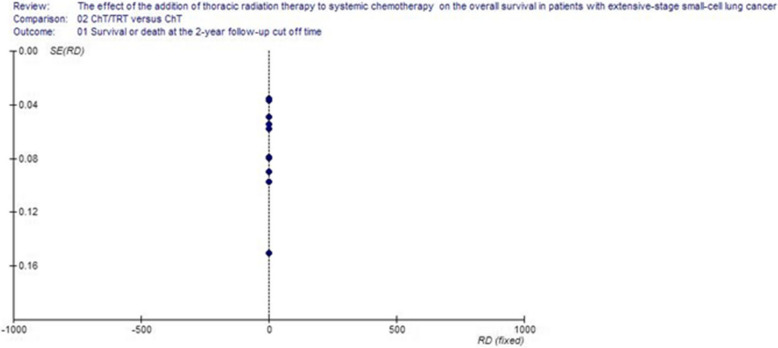
Funnel plot of the survival at the 2-year follow-up cut-off time regarding the risk difference

**Fig. 10:**
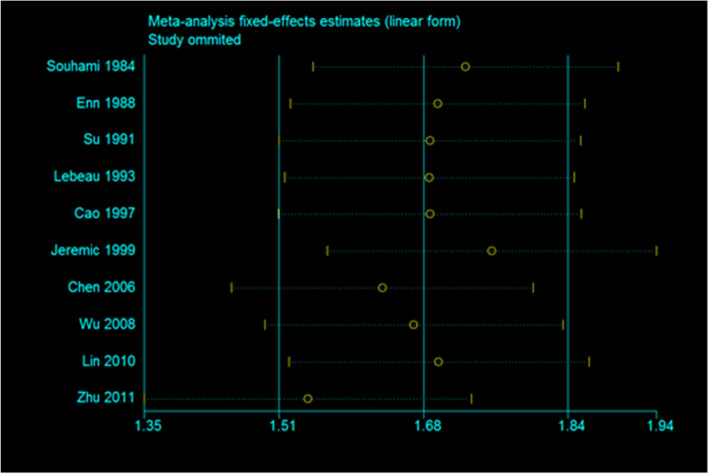
The sensitivity analysis of 1-year survival regarding the RR

**Fig. 11:**
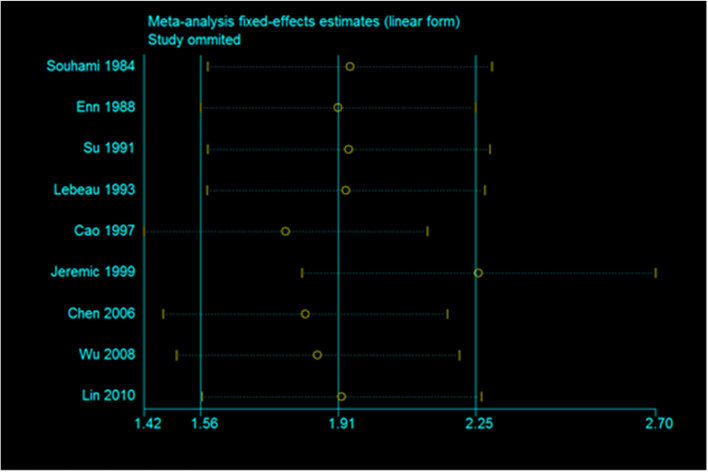
The sensitivity analysis of 2 -years survival regarding the RR

The following data were extracted from the included studies: journal name, year of publication, names of the authors, study type (retrospective or prospective), sample size, number of patients in the treatment and control groups, number of patients in each group at one and 2 years, dose of radiation therapy administered, and total number of patients alive at 1 year and 2 years. Data extraction was performed by the author. The primary clinical outcomes were 1- and 2-year overall survival (Figs. 1, 2, 3, 4, 5, 6, 7, 8, 9 ,10 and 11).

### Statistical analysis

Relative risk (RR) and treatment difference (RD) were the endpoints, and both of these parameters were used to assess treatment efficacy. The Mantel‒Haenszel method was used to determine the relative risk. The overall patient count and total patient survival at the 1- and 2-year follow-ups for both groups were determined based on the published data.

In this meta-analysis, Cochran’s *Q* statistic was used to quantify heterogeneity, which was considered significant at *P* < 0.05. The *I*^2^ statistic was used to assess heterogeneity regardless of the number of studies. When there was no significant heterogeneity, a fixed effects model was used; otherwise, a random effects model was used.

A sensitivity analysis was also performed to assess the influence of each individual study on the overall estimates via the sequential removal of individual studies. Publication bias was estimated via a funnel plot. Statistical analyses were carried out using Review Manager (RevMan; version 4.2 for Windows. Copenhagen: The Nordic Cochrane Centre, the Cochrane Collaboration, 2003) and Stata (Stata 10.0).

Relative risk is defined as the ratio of the probability of that event occurring in the exposed group to the probability in the nonexposed group [8–10]:

When the relative risk is equal to 1, there is no difference in risk between the two groups. When the relative risk is less than 1, the event is less likely to occur in the treatment group than in the control group. When the relative risk is greater than 1, the event is more likely to occur in the treatment group than in the control group. The risk difference (RD) is defined as the difference between the observed risks and reflects the actual difference in risk between the treatment group and the control group. If the risk is reduced, the RD is less than 0; if the risk increases, the RD is greater than 0. The RD ranges from − 1 to 1.

## Results

### Identification of studies

The literature search yielded 49 relevant articles comparing 1-year and 2-year overall survival rates among individuals with advanced-stage small-cell lung cancer. Thirty-nine studies were excluded from the analysis; thus, 10 studies were ultimately included for meta-analysis. There were several reasons for exclusion: some papers contained only limited data and were published in languages other than English or Chinese (*n* = 31); some did not mention overall survival in either the treatment group or the control group (*n* = 7); and one compared not only the effects of chemotherapy and thoracic radiation therapy but also the use of other kinds of treatments (*n* = 1).

The 10 eligible studies in this meta-analysis included a total of 922 patients. The sample size ranged from 18 to 174 participants, with a median of 96 cases. Tables 1 and 2 present the overall number of patients in each study, as well as the total number of patients in the treatment and control groups. Four studies did not specify the type of study design. The other research was carried out prospectively.

**Table 1:** The 1-year overall survival in both treatment and control group

**Author and year of publication**	**Total number of patients**	**Total number of patients in the treatment group**	**Total number of patients in the control** **group**	**1-y**ea**r survival in the treatment group**	**1-y**ea**r survival in the control group**
Cao ka-jia et al. [11]	57	28	29	60.7%	36.8%
LIN Ping et al. 2010	117	44	73	14%	16%
WU Lina et al. [12]	68	40	28	52.5%	28.6%
Chen Dongfu et al. [13]	174	142	32	52.8%	28.9%
Su Mei et al. [14]	113	77	36	45.5%	27.7%
Huizhu [15]	119	60	59	80%	38%
Enn Nou [16]	54	28	26	32%	26%
Jeremic Branislav [17]	109	55	54	65%	46%
RL souhami 1984	93	50	43	45%	34%
B Lebeau [8]	18	10	8	10%	25%

**Table 2:** The 2 years overall survival in both treatment and control group

**Author and year of publication**	**Total number of patients**	**Total number of patients in the treatment group**	**Total number of patients in the control** **group**	**2-y**ea**r survival in the treatment group**	**2-y**ea**r survival in the control group**
Cao ka-jia et al. [11]	57	28	29	27.6%	7.4%
LIN Ping et al. 2010	117	44	73	4.5%	2.7%
WU Lina et al. [12]	68	40	28	20%	7.14%
Chen Dongfu et al. [13]	174	142	32	19.7%	7.8%
Su Mei et al. [14]	113	77	36	7.8%	5.5%
Huizhu [15]	119	60	59	35%	26.1%
Enn Nou [16]	54	28	26	24%	12.9%
Jeremic Branislav [17]	109	55	54	38%	28%
RL souhami 1984	93	50	43	10%	8%
B Lebeau [8]	18	10	8	10%	12%

### Tests for heterogeneity of study

In this meta-analysis, there was acceptable heterogeneity among the studies for 1-year (*P* = 0.07, *I*^2^ = 44%) and 2-year survival (*P* = 0.04, *I*^2^ = 49.2%) were used. Therefore, the fixed effects model was used to calculate pooled RRs and RDs.

### Sensitivity analysis

In the sensitivity analysis, each study was omitted one at a time, and the pooled relative risk was recalculated again for the remaining studies to assess the stability of the meta-analysis results. The pooled RRs were similar before and after the elimination of each study, which means that the results of the meta-analysis were quite stable.

### Types of chemotherapy and radiotherapy dose

The majority of the chemotherapy regimens among the included studies involved either carboplatin and etoposide (CE) or cisplatin and etoposide (PE). The overall dose of TRT ranged from 40 to 60 Gy, with 1.8 to 2.0 Gy per fraction (Table 3).

**Table 3:** Chemotherapy regimens and radiotherapy doses of studies included

**Author and year of publication**	**Chemotherapy regimens used**	**T**horacic radiotherapy dose
Cao ka-jia et al. [11]	COA + CCNU^1^ EC + CCNU^2^	22–66 Gy
Zhu Hui 2011 [6]	CE-EP-	40–60 Gy
EnnNou [16]	CAV	40 Gy
Jeremic Branislav [17]	EP-EC	54 Gy
RL Souhami 1984	CAV	40 Gy
B Lebeau [8]	ECV	30–60 Gy
LIN Ping et al. 2010	EP	50–70 Gy
WU Lina et al. [12]	EP—CAV	50–70 Gy
Chen Dongfu et al. [13]	CE- CAP	20–60 Gy
Su Mei et al. [14]	COMVC	40–70 Gy

The prevalence of leukopenia was significantly greater in the ChT/TRT group. The prevalence rates of other toxicities did not differ significantly between the groups. Patients who received TRT experienced acute responses such as oesophagitis and radiation pneumonitis.

There were no significant differences in survival based on the drugs used for chemotherapy.

### Statistical results

Overall survival data were available for 922 patients across the ten included studies, with 534 patients in the treatment group (i.e., the chemotherapy plus thoracic radiation therapy (ChT/TRT) group) and 388 patients in the control group (i.e., the chemotherapy (ChT) group).

In the treatment group, 278 patients were alive at the 1-year follow-up, and 100 patients were alive at the 2-year follow-up. The 1-year overall survival rate in the treatment group (ChT/TRT group) was 52%, and the 2-year overall survival rate was 18.7%.

In the control group (ChT group), 125 patients were alive at the 1-year follow-up, and 39 patients were alive at the 2-year follow-up.

The 1-year overall survival rate in the control group (ChT group) was 32.2%, and the 2-year overall survival rate was 10%.

There was no heterogeneity among studies in terms of the pooled RRs for 1-year overall survival (*P* = 0.69, *I*^2^ = 0.0%) or 2-year overall survival (*P* = 0.85, *I*^2^ = 0.0%), and there was acceptable heterogeneity among studies in terms of the pooled RDs for 1-year overall survival (*P* = 0.07, *I*^2^ = 44%) and 2-year overall survival (*P* = 0.04, *I*^2^ = 49.2%). The RRs and RDs for both 1-year and 2-year overall survival were pooled using a fixed effects model.

The 1-year survival rate of the treatment group (ChT/TRT group) was significantly better than that of the control group (ChT group), with a pooled RR of 1.61 (95% CI, 1.36–1.90, *P* < 0.00001) and a pooled RD of 0.2 (95% CI, 0.13–0.26, *P* < 0.00001). The 2-year survival was significantly better in the ChT/TRT group than in the ChT group, with a pooled RR of 1.90 (95% CI, 1.34–2.68, *P* = 0.0003) and a pooled RD of 0.09 (95% CI, 0.05–0.13, *P* < 0.0001).

Sensitivity analysis was conducted via the leave-one-out approach. The pooled RRs were similar before and after the elimination of each study, indicating that the results were robust.

Publication bias was analyzed using funnel plots and Egger’s test. There was no publication bias for 1-year overall survival (*P* = 0.166) or 2-year overall survival (*P* = 0.285).

## Discussion

Since extensive-stage small cell lung cancer is not thought to be curable, the goals of treatment are to reduce symptoms and increase the survival rate. Most doctors believe that thoracic radiation therapy should be used only for severe symptoms such as pain and bleeding and that adding thoracic radiation therapy to chemotherapy can increase the risk of severe side effects (e.g., pneumonia or difficulty swallowing and eating). Therefore, it is best to consider this combination approach only when necessary [18].

According to the National Comprehensive Network of Cancer (NCCN), thoracic radiation therapy is only used for treating patients with bulky tumors or distant metastatic symptoms. In many studies, the addition of thoracic radiation therapy has demonstrated a clear overall survival advantage. Thoracic radiation therapy is always used in combination with chemotherapy as palliative treatment in patients with advanced-stage small-cell lung cancer. In addition to increasing the survival rate, thoracic radiation therapy combined with chemotherapy decreases the likelihood that thoracic tumors will recur. Although treatment approaches for small cell lung cancer have changed over the past 30 years, the results in clinical practice are still rather diverse [19–22].

Among the 10 studies included in this meta-analysis, 8 studies revealed that combination therapy had a significant positive effect on 1-year overall survival, whereas the remaining 2 studies revealed that 1-year overall survival rate was better when chemotherapy alone was used. Nine studies reported that 2-year overall survival was better when administering combination therapy, whereas one study reported that 2-year overall survival was better when administering chemotherapy alone.

This meta-analysis aimed to elucidate the effects of adding thoracic radiation therapy to chemotherapy among patients with advanced-stage small-cell lung cancer. Ten studies involving 922 patients were included in this analysis. The included studies were published in either Chinese or English. A total of 534 patients were included in the ChT/TRT group, and 388 patients were included in the control group. In the ChT/TRT group, the 1-year and 2-year overall survival rates were 52% and 18.7%, respectively, whereas in the ChT group, the 1-year and 2-year overall survival rates were 32.2% and 10%, respectively. These results show that adding thoracic radiation therapy to systemic chemotherapy improves the survival of patients with advanced-stage small-cell lung cancer.

Since limited data were provided, the role of adding thoracic radiation therapy in the local control of the disease in the chest could not be determined. Most of the included studies did not examine the progression-free survival rate of a recurrence in the thorax or a distant area. Hui Zhu reported that the 2-year PFS was 11.1% for the whole cohort and that the 2-year PFS in the treatment group (ChT/TRT group) was 12.6%, whereas the 2-year PFS in the control group (ChT group) was 7.2%. Jeremic et al. [17] reported that local control and 2-year overall survival were better in patients receiving thoracic radiation therapy, but there was no difference in distant metastasis control. Zhu Hui et al. (2011) reported that 1-year overall survival was greater than 80% in the treatment group and 38% in the control group when regimens of EC-EP with radiation doses ranging from 40–60 Gy were used. Therefore, the best outcome for both 1-year and 2-year overall survival is the use of EC‒EP regimens in combination with 40‒60 Gy radiation therapy.

In terms of side effects, most of the studies mentioned that there were no differences in the incidence of vomiting, nausea, or anorexia. However, leukopenia, oesophagitis, and pneumonitis are the most common side effects in patients treated with ChT/TRT.

Some studies have shown that the benefit of radiotherapy is greater in young patients and that toxicity is greater in older patients. Because of the limited amount of available data, it is difficult to determine this benefit, and we were unable to assess 5-year overall survival for the same reason. Jeremic [17] reported that the 5-year overall survival (OS) of patients treated with radiotherapy plus chemotherapy was 9.1%, whereas that of patients treated with chemotherapy alone was 3.7%.

This meta-analysis has several limitations, such as the inclusion of studies with a very small sample size (18 patients), which may have biased the results of this study. Additionally, there were no restrictions regarding the age of the patients, KPS, brain metastasis, weight loss, the presence of malignant pleural effusion, whether the patients received chemotherapy concurrently, the site of distant metastasis, or whether the patients received PCI.

## Conclusion

According to this meta-analysis, the 1-year overall survival rate increases to 52% when thoracic radiation therapy is added to systemic chemotherapy compared with 32.2% when chemotherapy is used alone. Additionally, the 2-year survival rate increased from 10% to 18.7% when chemotherapy was used alone.

The 1-year survival rate of the treatment group (ChT/TRT group) was significantly better than that of the control group (ChT group), with a pooled RR of 1.61 (95% CI, 1.36–1.90, *P* < 0.00001) and a pooled RD of 0.2 (95% CI, 0.13–0.26, *P* < 0.00001). The 2-year survival of the ChT/TRT group was significantly better than that of the control group, with a pooled RR of 1.90 (95% CI, 1.34–2.68, *P* = 0.0003) and a pooled RD of 0.09 (95% CI, 0.05–0.13, *P* < 0.0001).

This meta-analysis provides more evidence that thoracic radiation increases overall survival in patients with extensive-stage small-cell lung cancer.

## Data Availability

Data is available within the paper. The authors affirm that the data supporting the study's conclusions are available in the journal.

## Abbreviations

TRT: Thoracic radiation therapy
BRT: Brain radiation therapy
RT: Radiation therapy
RR: Relative risk
RD: Risk difference
ES-SCLC: Advanced-stage small cell lung cancer
